# ASK1-Interacting Protein 1 Acts as a Novel Predictor of Type 2 Diabetes

**DOI:** 10.3389/fendo.2022.896753

**Published:** 2022-05-30

**Authors:** Zhigao Song, Cong Chen, Jipei He, Bixia Liu, Weidong Ji, Liangping Wu, Li He

**Affiliations:** ^1^ Center for Translational Medicine, The First Affiliated Hospital, Sun Yat-sen University, Guangzhou, China; ^2^ Department of Cardiovascular Surgery, Zhujiang Hospital of Southern Medical University, Guangzhou, China; ^3^ Department of Metabolic Surgery, Your Doctor Medical Group, Guangzhou, China; ^4^ Department of Metabolic Surgery, Jinshazhou Hospital of Guangzhou University of Traditional Chinese Medicine, Guangzhou, China; ^5^ Medical Research Center, Sun Yat-sen Memorial Hospital, Sun Yat-sen University, Guangzhou, China

**Keywords:** AIP1, visceral adipose, adipose inflammation, insulin resistance, type 2 diabetes

## Abstract

Type 2 diabetes (T2D) mellitus is a chronic inflammatory disease characterized with high secretion of tumor necrosis factor (TNF)-α, but the regulatory pathway of TNF-α production in T2D has not been fully elucidated. ASK1-interacting protein 1 (AIP1) is a signaling scaffold protein that modulates several pathways associated with inflammation. In this study, we aimed to investigate the role of AIP1 in T2D development. Our results revealed that AIP1 was downregulated in omental adipose tissue (OAT) of obese patients with T2D compared with that in obese patients. In addition, Pearson’s correlation test showed that AIP1 was negatively correlated with the homeostatic model assessment for insulin resistance (HOMA-IR, r = -0.4829) and waist-to-hip ratio (r = -0.2614), which are major clinical indexes of T2D. As revealed by the proteomic analysis, immunohistochemistry, and ELISA, the OAT and the serum of obese patients with T2D presented high inflammatory status. And the increased inflammatory factors TNF-α and C-reactive protein C (CRP) in the serum of obese patients with T2D showed a positive correlation with HOMA-IR (TNF-α, r = 0.4728; CRP, r = 0.5522). Interestingly, AIP1 deficiency in adipocytes facilitated TNF-α secretion and retarded glucose uptake. Mechanistically, *AIP1* deletion in human adipocytes activated JNK, p38 MAPK, and ERK1/2 signaling. Furthermore, inhibition of these signaling pathways using specific inhibitors could suppress these signal activation and insulin resistance caused by AIP1 deficiency. In addition, AIP1 and TNF-α expression in the OAT of patients with T2D recovered to normal levels after laparoscopic Roux-en-Y gastric bypass (RYGB) surgery. These findings indicate that AIP1 is negatively correlated with the clinical indexes of T2D. It modulates TNF-α expression in OAT *via* JNK, p38 MAPK, and ERK1/2 signaling.

## Introduction

Diabetes is a life-threatening disease that occurs when the body cannot produce sufficient insulin or effectively utilize it. The International Diabetes Federation (IDF) (https://www.diabetesatlas.org) estimated that there are 463 million people with diabetes worldwide, and type 2 diabetes mellitus (T2D) accounts for the overwhelming majority (approximately 90%) of diabetes cases, with approximately 11.3% of deaths due to diabetes and its complications. Cardiovascular diseases are reportedly associated with one-third to half of all diabetes-related deaths. However, the mechanisms underlying diabetes, especially T2D, require further investigation.

It is established that obesity is an independent risk factor for T2D (1, 2). Interestingly, visceral adipose tissue (VAT), rather than subcutaneous adipose tissue, plays an indispensable role in the onset of T2D (3, 4). Compared with subcutaneous adipose tissue, VAT is frequently in a state of chronic and low-grade inflammation during obesity (5). Concurrently, macrophages accumulate in the adipose tissue and are responsible for the secretion of inflammatory factors, including tumor necrosis factor (TNF)-α, monocyte chemotactic protein (MCP)-1, and interleukin 6 (IL-6) (6). Specifically, TNF-α is particularly important in patients with T2D. TNF-α deficiency in mice results in significantly improved insulin sensitivity (7). In addition, a lack of TNF-α receptors leads to obesity-associated insulin resistance (8). However, it is still unclear whether the recruited macrophages or inflamed adipocytes are overriding cells that secrete cytokines in inflamed human VATs. Recent studies have focused on the effect of inflammatory factors derived from infiltrating macrophages or total inflammatory factors on T2D (6, 9, 10). Nevertheless, the role of adipocytes in VAT on inflammatory factor expression warrants further studies.

ASK1-interacting protein-1 (AIP1), also known as DAB2-interacting protein-1 (DAB2IP), is a member of the Ras GTPase-activating protein family (11). RAS-GTP superfamily members are reportedly related to T2D (12). Previous studies reveal that AIP1 modulates several signaling pathways induced by TNF-α. TNF-α can lead to AIP1 unfolding, resulting in the interaction of AIP1 and ASK1 (11). AIP1 enhances ASK1-JNK apoptotic signaling by facilitating TNF-α-induced de-phosphorylation of ASK1 at pSer967 (13). AIP1 is associated with TNF receptor associated factor 2 (TRAF2) and inhibits TNF-α-induced NF-κB activation (14). Moreover, studies have shown that AIP1 deletion in mice enhances inflammatory responses in ischemic hindlimb (15), inflammatory sponge (16), carotid ligation (17), atherosclerosis (18), and graft arteriosclerosis models (12). Owing to its anti-inflammatory function and its relationship with TNF-α, we hypothesized that AIP1 might be involved in the development of T2D.

The role of AIP1 in the VAT of patients with T2D has not yet been determined. However, in the present study, we found that AIP1 expression was downregulated in the visceral adipocytes of patients with T2D, and it had a strong clinical relevance with insulin resistance. Furthermore, AIP1-deficient adipocytes displayed higher TNF-α production and impaired insulin signaling. Our results suggest that lower adipocyte AIP1 expression can act as a novel predictor of T2D by facilitating TNF-α production and downregulating the insulin pathway.

## Materials and Methods

### Reagents and Antibodies

Human adipose-derived stem cells (ADSCs) (HUXMD-01001) and adipogenic differentiation medium (HUXMD-90031) were obtained from Cyagen (Santa Clara, California, USA). TNF-α (C008) was purchased from Novoprotein (Shanghai, China). Anti-DAB2IP (ab87811), TNF-α (ab6671), and β-tubulin (ab6046) antibodies, Glucose Uptake Assay Kit (ab136955) were purchased from Abcam (Cambridge, UK). DAB Substrate Kit (8059S), and IHC Detection Reagent (8125S, 8114S), anti-phospho-SAPK/JNK (9255S), SAPK/JNK (9252S), Phospho-p38 MAPK (Thr180/Tyr182) (9211S), p38 MAPK(8690S), phospho-p44/42 MAPK (Erk1/2) (Thr202/Tyr204) (9106S), p44/42 MAPK (Erk1/2) (4695S), and rabbit IgG (2729S), HRP-linked (7074S) antibodies were purchased from Cell Signaling Technology (Danvers, Massachusetts, USA). Antifade mountant (P36934), Bicinchoninic acid (BCA) Protein Assay Kit (23225), BODIPY (D3922) and Hoechst (H21486) were purchased from Invitrogen (Waltham, Massachusetts, USA). TNF-α (EK0525) and C-reactive protein (CRP) (EK1316) ELISA kits were purchased from BOSTER (Wuhan, China). SB203580 (S1076), KO-947 (S8569), and SP600125 (S1460) were purchased from Selleck (Houston, Texas, USA). LentiCRISPRv2, VSVG, RA2, TAT, and HEPM2 plasmids were purchased from Addgene (Watertown, Massachusetts, USA). Glucose Assay Kit (Glucose oxidase and Peroxidase (GOD-POD) Method) (BC2505) were purchased from Solarbio (Beijing, China). Radio-immunoprecipitation assay buffer (RIPA) buffer (P0013B), Hematoxylin and Eosin (HE) Staining Kit (C0105S), Citrate Antigen Retrieval solution (P0083), Puromycin (ST551) were purchased from Beyotime (Shanghai, China). Polyvinylidene difluoride (PVDF) (IPVH00010) membranes and enhanced chemiluminescence (ECL) (WBKLS00100) substrates were purchased from Merck Millipore (Burlington, Massachusetts, USA). TMT-reagent (Art.No.90111) were purchased from ThermoFisher Scientific (Waltham, Massachusetts, USA).

### Recruitment

46 obese patients and 46 obese patients with T2D who underwent laparoscopic sleeve gastrectomy or laparoscopic Roux-en-Y gastric bypass (RYGB) surgery were recruited from Jinshazhou Hospital of Guangzhou University of Traditional Chinese Medicine during July 2019 to October 2020. All subjects were of Asian. As recommended by the Working Group on Obesity in China (19), subjects were categorized by body mass index (BMI) ≥ 28 kg/m^2^ as obese. As recommended by Chinese Diabetes Society, subjects were categorized by Diagnostic Criteria for Type 2 Diabetes launched by WHO (1999) (20) as T2D. The obese participants didn’t suffer from T2D. All participants provided written consent, and the study was performed in accordance with the principles outlined in the Declaration of Helsinki. Ethical approval was obtained from the Jinshazhou Hospital of Guangzhou University of Traditional Chinese Medicine Ethics Committee (Reference Number, 19060101). Key eligibility criteria were male or female participants aged between 18 and 70 years who required bariatric surgery. Key exclusion criteria were any comorbidities such as acute infection, cancer, and any other consuming disease that could compromise the validity and safety of the study.

### Demographic and Clinical Features Collection

Demographic and clinical features were collected at the time of recruitment as described previously (21). Briefly, Weight was measured without shoes, bulky clothing, and accessories. Height was measured without shoes. BMI (kg/m^2^) was calculated as weight (kg) divided height (m) squared. Waistline (cm) referred as the perimeter of the plane formed by the top of the hip bone and the navel. Hipline (cm) referred as perimeter of the plane formed by widest part of hip. Waist-to-Hip Ratio (WHR) was calculated as Waistline (cm) divided Hipline (cm). After fasting overnight, fasting blood glucose (FBG) (mmol/L), glycated hemoglobin (HbA1c) (%), insulin (μU/mL), and connecting peptide (C-peptide) (ng/mL) were measured in clinical laboratory of Jinshazhou Hospital of Guangzhou University of Traditional Chinese Medicine. Homeostatic Model Assessment for Insulin Resistance (HOMA-IR) was calculated by FBG (mmol/L)×insulin (μU/mL)/22.5.

### Serum Samples Collection and Measurement of TNF-α and CRP Concentrations

After fasting overnight, pre-surgery blood samples were collected into blood collection tube containing ethylene diamine tetraacetic acid (EDTA), then centrifuged at 1000 g for 15 minutes. The serum was collected and stored at −80°C. Total serum TNF-α and C-reactive protein (CRP) was measured using an ELISA kit according to the manufactures protocol.

### Origin of Adipose Tissue

All subjects were of Asian origin. Omental adipose tissue (OAT) was obtained from 46 obese and 46 obese T2D patients who underwent laparoscopic sleeve gastrectomy or laparoscopic RYGB surgery. In addition, 3 of the 46 obese T2D patients received other routine operations at 6 months or more post-RYGB surgery due to other physical discomfort. During the 1st RYGB surgery, and the 2nd routine surgery, we obtained their OAT specimens.

### Proteomic Sequencing

Briefly, proteins were extracted from OAT of obese patients or obese patients with T2D, and protein concentration was determined using BCA Protein Assay Kit. After reduction, cysteine alkylation, and digestion, the samples were labeled with TMT-reagent. After C18 solid-phase extraction desalting, peptides were used for nano-liquid chromatography-mass spectrometry/mass spectrometry (LC–MS/MS) analysis using Q Exactive Plus quadrupole orbitrap mass spectrometer (Thermo fisher). The MS proteomics data have been deposited to the ProteomeXchange Consortium *via* the PRIDE (22) partner repository with the dataset identifier PXD033235. Next, the expressed proteins were identified. A fold change threshold (>1.2 or <0.83) was used to identify differentially expressed proteins. Gene Ontology (GO) (www.blast2go.com/b2ghome) was used to annotate all identified proteins. The mass spectrometry proteomics data have been deposited to the ProteomeXchange Consortium *via* the PRIDE partner repository with the dataset identifier PXD033235.

### Adipocyte Differentiation and Lipid Droplet Staining

When ADSCs reached approximately 100% confluence for 24 h, adipocyte differentiation was induced with ADSC adipogenic differentiation medium A for the first 3 days and ADSC adipogenic differentiation medium B for the next 2 days. After 15 days of this cycle, ADSC differentiation was verified by staining using BODIPY493/503. Briefly, the differentiated adipocytes were fixed with 4% paraformaldehyde for 12 minutes at room temperature. Then incubated with PBS containing 2 μmol/L BODIPY493/503 and 5 μg/mL Hoechst for 10 min. Cells were mounted with antifade mountant. The fluorescence microscope (IX71, Olympus) was used to capture the specific signals of six randomly selected, non-overlapping fields. Image J (Fiji) with the plugin Adiposoft (23) was used to measure the diameters(μm) of lipid droplet size, and the lipid stain percent (%, characterized by the percentage of BODIPY positive cells). This evaluation has been performed blindly by two independent individuals. The differentiated adipocytes are defined as cells whose cytoplasm is filled with lipid droplets. Only cultures with >70% adipocytes were used in further experiments. The differentiated adipocytes were maintained with adipogenic differentiation medium B.

### Lentivirus Package and Infection

The endogenous AIP1 was knocked-down by clustered regularly interspaced short palindromic repeats/clustered regularly interspaced short palindromic repeat–associated 9 (CRISPR/Cas9) in human ADSCs. The sequences of sgRNA of AIP1 were as followed: 5’-3’: caccgAAGTACCTGCAGGACGCCCT, aaacAGGGCGTCCTGCAGGTACTTc, targeting AIP1 exons were inserted into lentiCRISPRv2 vector, respectively. Non-targeting sgRNA (sgRNA-Ctrl) sequences were inserted into lentiCRISPRv2 vector as negative control. Thereafter, we packaged LentiCRISPRv2 vector with VSVG, RA2, TAT, and HEPM2 plasmid to generate lentivirus (negative control, LV-sgRNA-Ctrl; to knockout AIP1, LV-sgRNA-AIP1) and infected ADSCs. Puromycin (1.5 ug/mL) is used to screen stable AIP1 knockout and negative control ADSCs.

### *In Vitro* Supernatant Glucose, TNF-α Level, and Adipocytes 2-Deoxy-D-Glucose (2-DG) Uptake Measurement

The ADSCs were infected with LV-sgRNA-AIP1 or LV-sgRNA-Ctrl. Then, the ADSCs were differentiated to matured adipocytes. The matured adipocytes were treated with TNF-α (10 ng/mL). And the supernatant was collected at 4, 8, 12, and 24 h after treatment.

The supernatant glucose concentration was measured using Glucose Assay Kit (GOD-POD Method) following the manufacturer’s instructions.

For TNF-α measurement, *in vitro* cultured adipocytes were pre-treated with TNF-α (10 ng/mL) for 4, 8, 12, and 24 hours and supernatant was replaced with serum-free medium, and the medium was collected after 24 hours. ELISA assays for TNF-α was performed following the manufacturer’s instructions.

Glucose Uptake Assay Kit was used to evaluate the 2-DG uptake of matured adipocytes. Briefly, ADSCs infected with LV-sgRNA-AIP1 or LV-sgRNA-Ctrl were seeded into 96 well plate and differentiated to adipocytes. Matured adipocytes were pre-treated with or without TNF-α (10 ng/mL) for 12 hours and then starved with serum free medium overnight, followed by pre-incubating with 100 µL Krebs-Ringer-Phosphate-HEPES (KRPH) Buffer (20 mM HEPES, 5 mM KH_2_PO_4_, 1 mM MgSO_4_, 1 mM CaCl_2_, 136 mM NaCl, and 4.7 mM KCl, pH 7.4) containing 2% Bovine serum albumin (BSA) for 40 minutes. Then, 10 µl of 2-DG (10 mM) was added into the KRPH Buffer and incubated for 30 minutes at 37°C. And the 2-DG uptake was measured following the manufacturer’s instructions.

For the inhibitor experiments, the ADSCs were infected with LV-sgRNA-AIP1 or LV-sgRNA-Ctrl. Then, the ADSCs were differentiated to matured adipocytes and pre-treated with specific inhibitors, including 10 μM p38 MAPK inhibitor SB203580, 10 μM ERK1/2 inhibitor KO-947, and 10 μM JNK inhibitor SP600125 for 1 hour. And the adipocytes were further stimulated by 10 ng/mL TNF-α combined with the specific inhibitors for 12 hours. The supernatant glucose concentration was analyzed using Glucose Assay Kit. Further, supernatant was replaced with serum-free medium and incubated for another 24h, TNF-α concentration in the serum-free medium was analyzed using ELISA kit. For 2-DG uptake measurement, cells seeded in 96 well plate were starved overnight with serum free medium, then pre-incubated with 100 µL KRPH buffer containing 2% BSA for 40 minutes. And followed by adding 10 µL 2-DG (10 mM) into the KRPH Buffer and incubating for 30 min at 37°C. Glucose Uptake Assay Kit was used to measure the 2-DG uptake.

### Western Blotting

Proteins were extracted from adipocytes in radio-immunoprecipitation assay (RIPA) lysis buffer. Protein concentrations were evaluated using a BCA Protein Assay Kit. Samples with the same protein quantity were separated using SDS-PAGE and transferred onto polyvinylidene difluoride membranes. The membranes were incubated with primary antibodies against AIP1, JNK, p-JNK, Erk1/2, p-Erk1/2, P38, p-P38, and β-tubulin, and then with the appropriate HRP-conjugated secondary antibodies, the specific signals were detected by iBright CL1500 Imaging System (Invitrogen) using ECL substrates. And the quantification analyses were accessed by using the Image J software (Fiji).

### Immunohistochemistry (IHC), Hematoxylin and Eosin (HE) Staining and Adipocyte Cell Size Determination

OAT was isolated from the omentum majus of obese or obese T2D patients who underwent laparoscopic sleeve gastrectomy or laparoscopic RYGB surgery and fixed with 4% paraformaldehyde for 24 h, paraffin-embedded, and cut into 5-μm-tick sections.

For immunohistochemistry, six serial sections of each tissue were deparaffinized, rehydrated, retrieved at 95°C for 20 minutes using Citrate Antigen Retrieval solution and cooled down to room temperature. After blocking endogenous peroxidase activity with 3% H_2_O_2_ in methanol for 12 minutes, washing thrice with PBS, and blocking with 10% goat serum for 1 hour, the slides were incubated with primary antibodies (rabbit anti-AIP1, 0.5 μg/ml; rabbit anti-TNF-α, 2.5 μg/ml) at 4°C overnight, the same concentration of rabbit IgG antibody was used as negative control. Then washed thrice with PBS and incubated with secondary antibodies. Finally, the DAB and hematoxylin staining were used to detect the specific signals and the nucleus. After dehydration by using increasing concentrations of ethanol (70%, 80%, 90% and 100%) and xylene, the sections were mounted with neutral balsam and the specific signals were captured using AxioImagerZ2 Microscope (Zeiss).

For HE staining, six serial sections of each tissue were deparaffinized, rehydrated, and conducted using the Hematoxylin and Eosin Staining Kit according to the manufactures protocol.

Image J (Fiji) was used to measure the mean intensity of positive signals of six randomly selected, non-overlapping fields of each image. And the adipocyte size was measured by the Image J (Fiji) with the plugin Adiposoft (23). The staining intensity score was as follows: 0: negative, 1: weak; 2, moderate; 3, strong. The positive area was defined as follows: 0, <5%, 1: 5%–25%, 2: 26%–50%, 3: 51%–75%; and 4, >75%. The histochemical score (H-SCORE) was calculated by multiplying the score of the staining intensity and the score of the positive area. This evaluation has been performed blindly by two independent individuals.

### Statistical Analysis

Statistical analyses of clinical data were performed using SPSS software (Version 22.0). Other analyses were performed using GraphPad Prism 9.0 software (GraphPad Software, San Diego, California, USA). All Figures are representative of three experiments unless otherwise stated. Clinical data are presented as mean ± standard deviation (SD). Other data are presented as mean ± standard error of the mean (SEM). We firstly determined whether the data were normality using D’Agostino & Pearson omnibus normality test. For the normally distributed data, comparisons between two groups were performed using an unpaired Student’s *t*-test. Comparisons among more than two groups were performed using two-way ANOVA with Tukey’s multiple comparisons test. And Kruskal Wallis test was used for the abnormally distributed data. Pearson’s correlation test was used to analyze the correlation among multiple variables. *P*-values were two-tailed, and results with *P* < 0.05 were considered to indicate statistically significant (indicated with an asterisk *).

## Results

### AIP1 Was Negatively Correlated With Obesity-Related T2D

**Figure 1:**
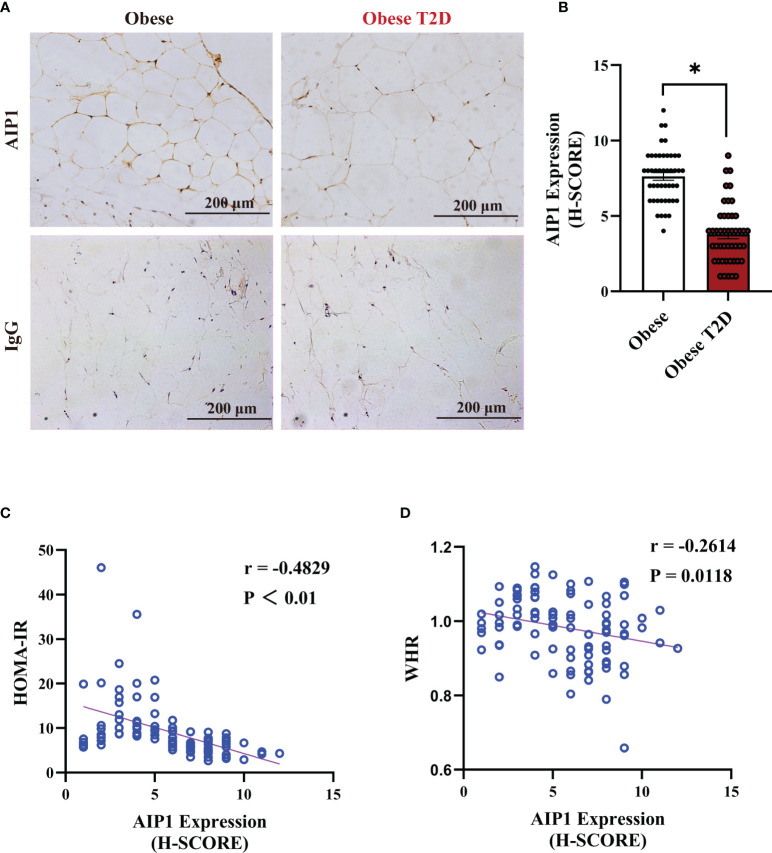
AIP1 was negatively correlated with obesity-related T2D. **(A, B)** Representative immune-histochemical staining **(A)**, and quantification **(B)** of AIP1 in the OAT of obese patients (n=46) and obese patients with T2D (n=46). Scale bar, 200 μm. Kruskal-Wallis test, *P < 0.05. **(C, D)** Pearson’s correlation test to reveal the correlations of AIP1 and HOMA-IR **(C)** or WHR **(D)** in 92 consecutively admitted patients with or without T2D, *P* < 0.05 indicated significant correlation.

To determine the relationship between AIP1 and T2D, we collected the OAT from 46 consecutively admitted patients with obesity and 46 consecutively admitted BMI-paired obese patients with T2D, recruited from clinics. Their baseline characteristics are shown in **Table 1**. Briefly, there were no differences in age, weight, and BMI between these groups. Furthermore, the characteristics related to T2D, such as waistline, WHR, FBG, HbA1c, and HOMA-IR, were significantly higher in obese patients T2D than in obese patient. We then assessed the expression of AIP1 in the OAT of obese patients with or without T2D. The results showed that the expression of AIP1 was significantly decreased in the OAT of obese patients with T2D (**Figures 1A, B**). Furthermore, Pearson’s correlation test was used to reveal the correlation between AIP1 expression and the baseline characteristics in these two groups. As shown in **Figures 1C, D**, AIP1 expression was negatively correlated with HOMA-IR (r = -0.4829) and WHR (r = -0.2614), which are major characteristics of T2D. These results indicated that the expression of AIP1 was negatively correlated with T2D.

**Table 1:** Demographics and clinical features of the 92 patients included in the study of AIP1 expression comparison.

Characteristics	Obese (n=46)	Obese T2D (n=46)	P*
**Demographic**			
Age, years	32.67 ± 8.48	34.54 ± 12.83	0.4118
Male, n (%)	28.26	56.52	/
Height (cm)	166.50 ± 7.50	168.80 ± 8.29	0.1655
Weight (kg)	102.30 ± 22.80	111.40 ± 25.40	0.0728
BMI (kg/cm^2^)	36.66 ± 6.54	38.88 ± 7.40	0.1309
Waistline (cm)	113.1 ± 15.05	119.9 ± 17.12	0.0446
Hipline (cm)	118.0 ± 11.54	119.1 ± 13.57	0.6787
WHR	0.9582 ± 0.08	1.007 ± 0.09	0.0063
**Disease status**			
FBG (mmol/L)	5.63 ± 0.91	8.36 ± 2.83	<0.0001
HbA1c (%)	5.96 ± 0.70	8.03 ± 1.70	<0.0001
Insulin (μU/mL)	26.95 ± 10.92	33.16 ± 19.82	0.0657
C-Peptide (ng/mL)	4.28 ± 4.28	3.89 ± 3.89	0.2272
HOMA-IR	6.88 ± 3.39	11.72 ± 7.88	0.0002

*Compared between patients with T2D and non-T2D obese patients using an unpaired Student’s t-test. Data are presented as mean ± SD.

BMI, body mass index; WHR, waist-to-hip ratio; FBG, fasting blood glucose; HOMA-IR, Homeostatic Model Assessment of Insulin Resistance.

### OAT of Patients With T2D Showed Increased Pro-Inflammatory Status

**Figure 2:**
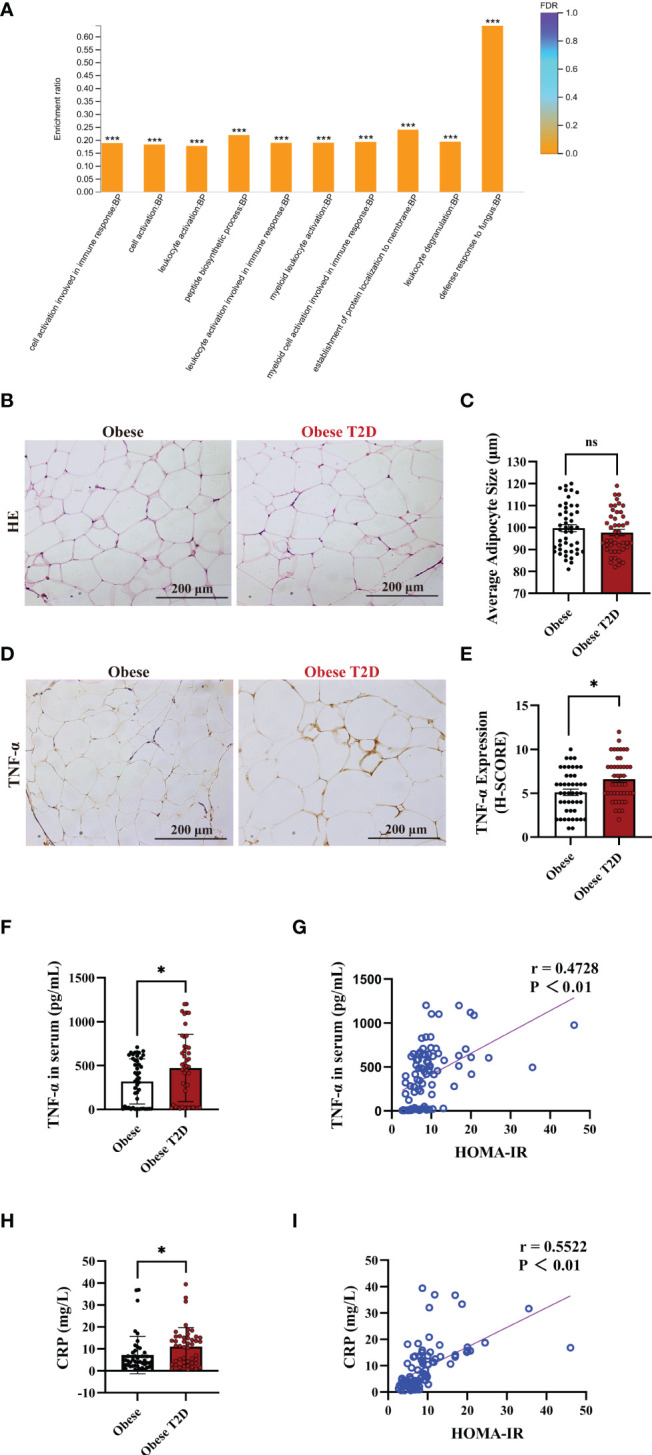
OAT of patients with T2D showed increased pro-inflammatory status. **(A)** Gene Ontology (GO) enrichment analysis of the biological process in the adipose tissue of obese patients and obese patients with T2D. The top ten biological processes were showed. Fisher’s Exact Test, ***FDR (FDR adjusted p-value) < 0.001. **(B, C)** Representative HE staining **(B)** and quantification of adipocyte size **(C)** in the OAT of obese patients and obese patients with T2D. Scale bar, 200 μm. Unpaired Student’s *t*-test, ns, no significance. **(D, E)** Representative immune-histochemical staining **(D)**, and quantification **(E)** of TNF-α in the OAT of obese patients and obese patients with T2D. Scale bar, 200 μm. Kruskal-Wallis test, *P < 0.05. **(F)** ELISA analysis of TNF-α in the serum of obese patients (n=46) and obese patients with T2D (n=46). Unpaired Student’s *t*-test *P < 0.05. **(G)** Pearson’s correlation test to reveal the corrections of TNF-α and HOMA-IR in 92 consecutive admission patients with or without T2D, *P* < 0.05 indicated significant correlation. **(H)** ELISA analysis of CRP in the plasma of obese patients (n=46) and obese patients with T2D (n=46). Unpaired Student’s *t*-test *P < 0.05. **(I)** Pearson’s correlation test to reveal the corrections of CRP and HOMA-IR in 92 consecutive admission patients with or without T2D, *P* < 0.05 indicated significant correlation.

To identify the regulating pathways of AIP1 in T2D, we performed an analysis using a proteomic array of OAT between obese patients with T2D and obese patients. The proteomic analysis indicated that obese patients with T2D presented a higher inflammatory response than obese patients (**Figure 2A**). TNF-α is one of the critical inflammatory factors related to T2D. Therefore, we further confirmed the expression of TNF-α in the OAT. The results revealed that there was no difference in the size of adipocytes between obese patients and obese patients with T2D (**Figures 2B, C**). However, TNF-α expression in the OAT of obese patient with T2D was significantly increased compared with that in the OAT of obese patients (**Figures 2D, E**). Then we accessed the concentration of TNF-α and CRP, two critical indexes of inflammation, in the serum of obese patients or obese patients with T2D using ELISA kit. Interestingly, the concentration of TNF-α and CRP was significantly increased in the serum of obese patients with T2D compared with that in obese patients (**Figures 2F, H**). Furthermore, Pearson’s correlation test revealed that the concentrations of TNF-α (**Figures 2G**) and CRP (**Figures 2I**) were positively correlated with HOMA-IR, respectively (TNF-α, r = 0.4728; CRP, r = 0.5522). These results suggest that the inflammatory status of OAT may be involved in the pathogenesis of T2D.

### *In Vitro* AIP1 Deletion Enhanced TNF-α Production and Insulin Resistance in Adipocytes

**Figure 3:**
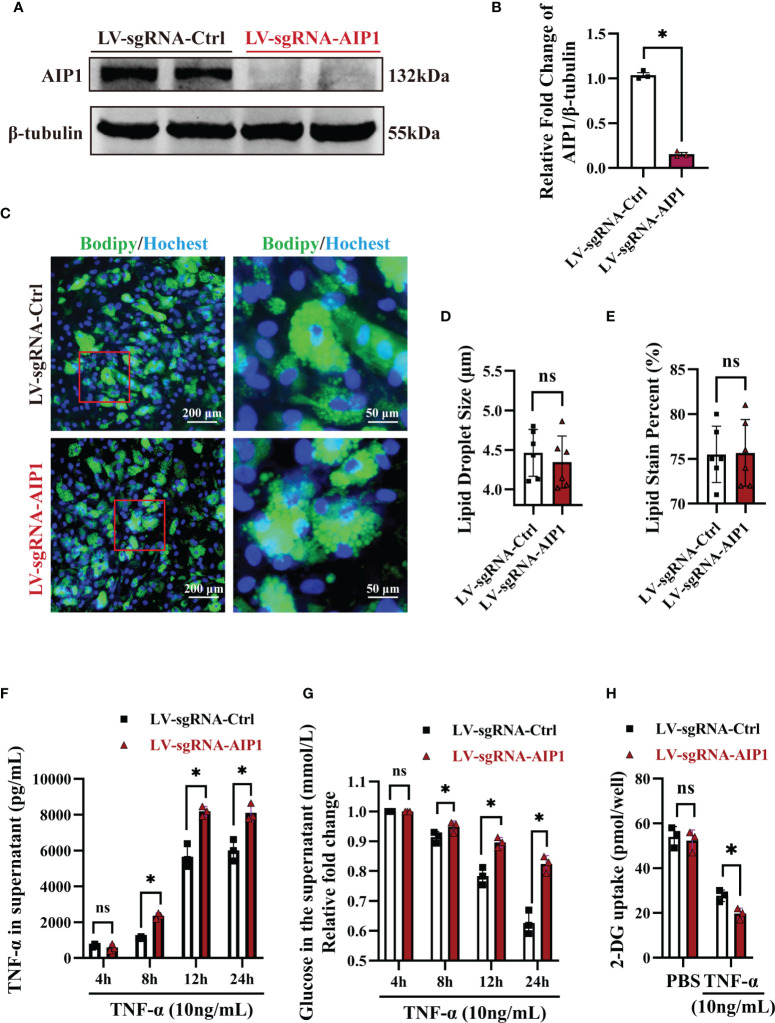
*In vitro* AIP1 deletion enhanced TNF-α production and insulin resistance in adipocytes. **(A, B)** Representative western blot analysis **(A)** and quantification **(B)** of AIP1 expression in adipocytes differentiated from ADSCs which were infected with LV-sgRNA-Ctrl or LV-sgRNA-AIP1, n=3, unpaired Student’s *t*-test, *P<0.05. **(C–E)** Representative immune-fluorescent images of lipids (green) stained by BODIPY **(C)**, the quantification of lipid droplet size **(D)**, and the quantification of lipid area per field **(E)** in human adipocytes differentiated from ADSCs which were infected with LV-sgRNA-Ctrl or LV-sgRNA-AIP1. The nuclei stained by Hoechst are indicated in blue. Scale bar, left, 200 μm; right, 50 μm; n=3, unpaired Student’s *t*-test, ns, no significance. **(F, G)** Human adipocytes differentiated from ADSCs which were infected with LV-sgRNA-Ctrl or LV-sgRNA-AIP1 and treated by 10 ng/mL TNF-α for 4, 8, 12, and 24 hours, the concentration of glucose **(G)** in the supernatant was determined using GOD-POD method. Further, supernatant was replaced with serum-free medium and incubated for another 24h, and the quantification of TNF-α in the supernatant **(F)** was performed using ELISA kit, n=3, two-way ANOVA followed by Tukey’s test for multiple comparisons, ns, no significance, *P < 0.05. **(H)** Human adipocytes differentiated from ADSCs which were infected with LV-sgRNA-Ctrl or LV-sgRNA-AIP1 and treated by 10 ng/mL TNF-α for 12 hours, then cells were starved overnight with serum free medium. Quantification of 2-DG uptake of adipocyte was performed using colorimetric method, n=3, two-way ANOVA followed by Tukey’s test for multiple comparisons, ns, no significance, *P < 0.05.

To investigate the role of AIP1 in TNF-α expression and T2D, AIP1 knockout (KO) human ADSCs were obtained using CRISPR-Cas9. ADSCs were differentiated into adipocytes, western blot analysis revealed that AIP1 was effectively knockout in adipocytes infected with LV-sgRNA-AIP1 **(** **Figures 3A, B**). And BODIPY was used to stain the differentiated adipocyte lipid droplets (**Figure 3C**). We found that there was no significant difference between AIP1-KO and vector-control adipocytes in the lipid droplet size (**Figure 3D**) and lipid staining percentage area (**Figure 3E**). Subsequently, we measured the concentration of TNF-α in the supernatant of differentiated adipocytes after TNF-α (10 ng/mL) stimulation at different time points. As expected, significantly higher TNF-α secretion (**Figure 3F**) was observed in the supernatant of AIP1 KO adipocytes. We then tested the glucose level in the cell supernatant at different time points after TNF-α (10 ng/mL) stimulation and found that compared with the control adipocytes, the AIP1 KO group had a higher sugar level in the supernatant (**Figure 3G**). The sugar uptake ability was decreased in AIP1 KO adipocytes compared with that in the control adipocytes after TNF-α (10 ng/mL) stimulation (**Figure 3H**). These results showed that the AIP1 deficiency in adipocytes resulted in less sugar consumption under TNF-α stimulation. The result also showed higher insulin resistance.

### AIP1 Modulated TNF-α Production and Insulin Resistance in Adipocytes *via* JNK and p38 MAPK/ERK Axis

**Figure 4:**
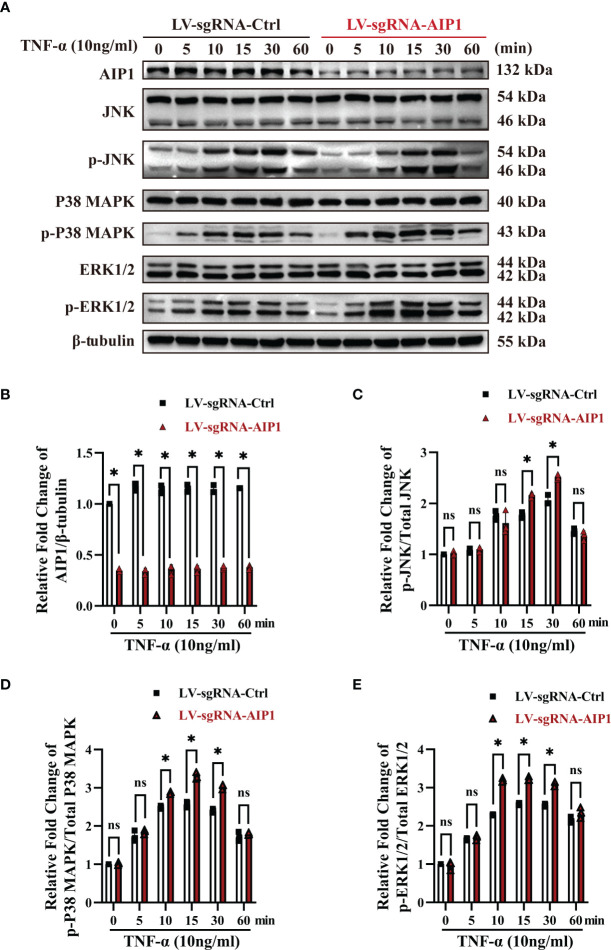
AIP1 deficiency in adipocytes promoted TNF-α induced JNK and p38 MAPK/ERK activation. **(A-E)** Representative western blot analysis and quantification of AIP1 **(A, B)**, p-JNK **(A, C)**, p-p38 MAPK **(A, D)**, and p-ERK **(A, E)** expression in human adipocytes differentiated from ADSCs which were infected with LV-sgRNA-Ctrl or LV-sgRNA-AIP1 and treated with 10 ng/mL TNF-α for 0, 5, 10, 15, 30, and 60 minutes, n=3, two-way ANOVA followed by Tukey’s test for multiple comparisons, ns, no significance, *P < 0.05.

**Figure 5:**
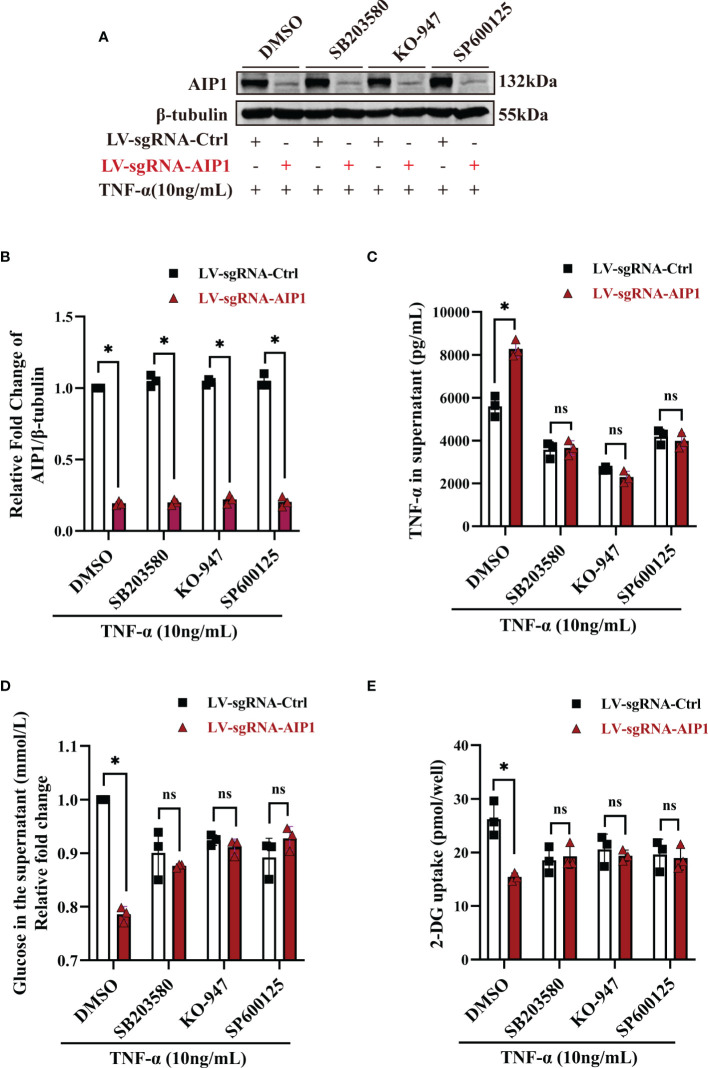
Inhibition of JNK, p38 MAPK and ERK reversed the changes caused by AIP1 deficiency in TNF-α treated adipocytes. ADSCs infected with LV-sgRNA-Ctrl or LV-sgRNA-AIP1 were differentiated to matured adipocytes and pretreated with specific inhibitors, including 10 μM p38 MAPK inhibitor SB203580, 10 μM ERK1/2 inhibitor KO-947, and 10 μM JNK inhibitor SP600125 for 1 hour. The adipocytes were further stimulated by 10 ng/mL TNF-α combined with the specific inhibitors for 12 hours. **(A, B)** The representative western blot analysis **(A)** and quantification **(B)** of AIP1, n=3, two-way ANOVA followed by Tukey’s test for multiple comparisons, *P < 0.05. **(C)** The supernatant was replaced with serum-free medium and incubated for another 24 hours, and the quantification of TNF-α in the supernatant was performed using ELISA kit, n=3, two-way ANOVA followed by Tukey’s test for multiple comparisons, *P < 0.05; ns, no significance. **(D)** The quantification of glucose in the supernatant was performed using the GOD-POD method, n=3, two-way ANOVA followed by Tukey’s test for multiple comparisons, *P < 0.05; ns, no significance. **(E)** Adipocytes were starved overnight with serum free medium. And the quantification of 2-DG uptake of adipocytes were performed using colorimetric method. n=3, two-way ANOVA followed by Tukey’s test for multiple comparisons, *P < 0.05; ns, no significance.

Previous research showed AIP1 modulated the inflammatory signaling including JNK and MAPK signaling. To further understand the molecular mechanism by which AIP1 modulates adipocyte TNF-α secretion and insulin resistance phenotype, the differentiated adipocytes were treated with TNF-α. As a result, AIP1 deletion caused enhanced TNF-α-induced activation of JNK, P38 and ERK1/2 in human adipocytes (**Figure 4**). Furthermore, suppression of P38 MAPK, ERK1/2 and JNK using their inhibitor SB203580, KO-947, and SP600125, respectively, abolished the secretion of TNF-α induced by AIP1 deficiency in TNF-α treated adipocytes (**Figures 5A–C**), and restored the glucose level in the cell supernatant (**Figure 5D**) and 2-DG uptake (**Figure 5E**) in the TNF-α treated AIP1-KO adipocytes. The results indicated that AIP1 modulated TNF-α production and insulin resistance in adipocytes *via* JNK and MAPK/ERK signaling.

### AIP1 Expression Was Increased After RYGB Surgery

**Figure 6:**
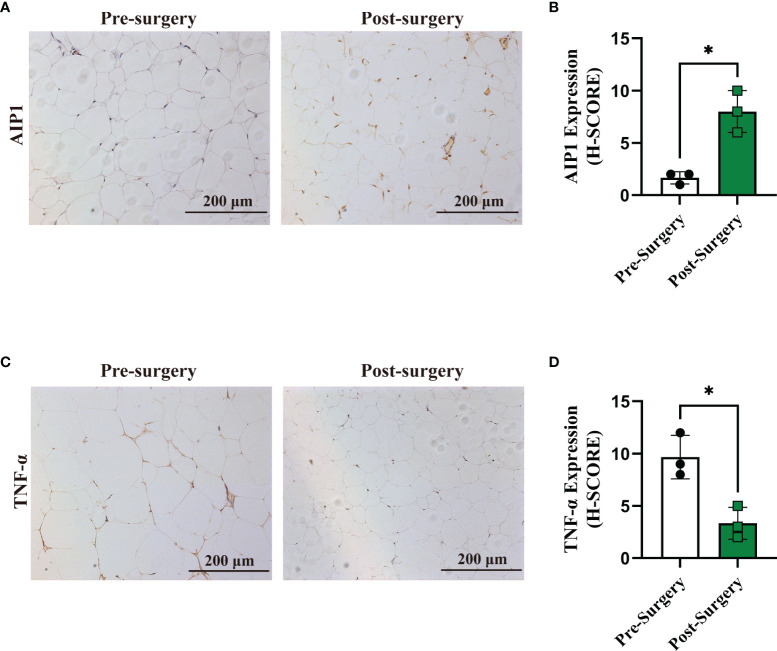
Expression of AIP1 was increased after RYGB surgery. **(A, B)** Representative immune-histochemical staining **(A)** and quantification **(B)** of AIP1 in the adipose tissues from patients with T2D who had undergone surgery (n=3) or not (n=3). Scale bar, 200 μm. Kruskal-Wallis test, *P < 0.05. **(C–D)** Representative immune-histochemical staining **(C)** and quantification **(D)** of TNF-α in the adipose tissues from patients with T2D who had undergone surgery (n=3) or not (n=3). Scale bar, 200 μm. Kruskal-Wallis test, *P < 0.05.

It was well known that metabolic surgery could considerably improve the diabetic state and metabolic syndrome of patients. Therefore, we collected the OAT from patients with T2D pre- and post-surgery (**Table 2**). After surgery, all of the characteristics related to patients with T2D, including weight, BMI, waistline, WHR, FBG, HbA1c, and HOMA-IR, were significantly decreased in obese T2D patients. We then performed IHC staining and found that compared with that in pre-surgery, the expression of AIP1 in adipose tissue after metabolic surgery was increased (**Figures 6A, B**), accompanied by lower TNF-α expression (**Figures 6C, D**). Taken together, these findings suggested that AIP1 is an integral part of the feedback loop of low-grade inflammation in OAT of patients with T2D.

**Table 2:** General clinical characteristics of followed-up patients.

	Pre-surgery (n=3)	Post-surgery (n=3)
Weight (kg)	116.97 ± 14.83	93.23 ± 11.58*
BMI (kg/cm^2^)	40.41 ± 5.65	32.15 ± 4.07*
Waistline(cm)	124.00 ± 15.56	105.33 ± 13.89*
WHR	0.96 ± 0.08	0.93 ± 0.08*
FBG (mmol/L)	10.81 ± 0.73	5.33 ± 0.51*
HbA1c(%)	10.23 ± 2.38	5.47 ± 0.33*
HOMA-IR	10.33 ± 1.17	3.26 ± 0.26*

*P < 0.05, compared between pre-surgery and second-surgery patients using an unpaired Student’s t-test. Data are presented as mean ± SD.

BMI, body mass index; WHR, waist-to-hip ratio; FBG, fasting blood glucose; HOMA-IR, Homeostatic Model Assessment of Insulin Resistance.

## Discussion

**Figure 7:**
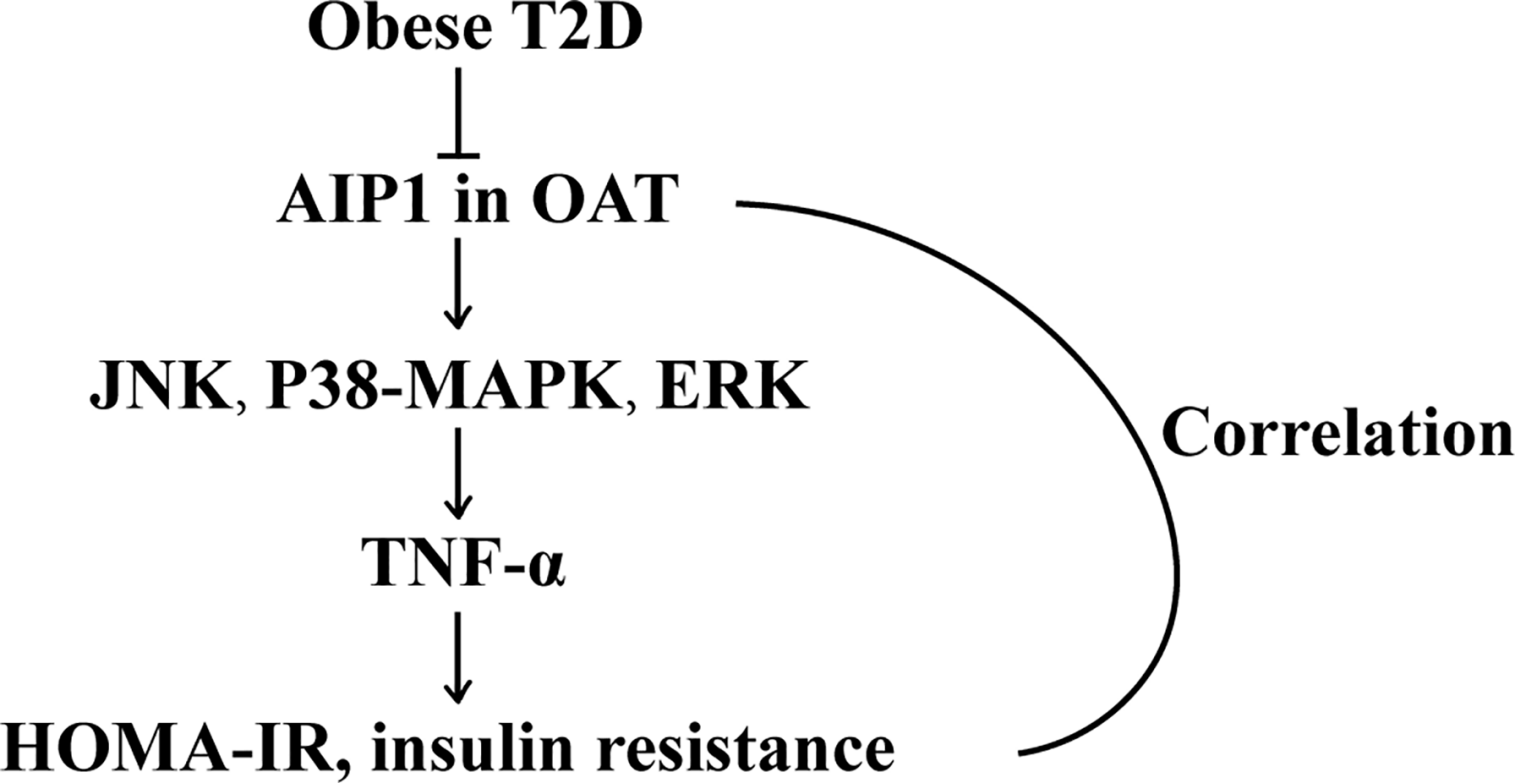
The schematic diagram of the role of AIP1 in T2D. AIP1, a novel member of the Ras-GAP family, was decreased in the OAT of T2D patients. Furthermore, AIP1 expression negatively correlated with HOMA-IR and WHR, the major features of patients with T2D. Mechanistically, AIP1 deficiency in the OAT elevated the expression of inflammatory factors *via* JNK/p38 MAPK/ERK signaling.

In the current study, we demonstrated that AIP1 was considerably decreased in the OAT of obese patients with T2D compared with that in the obese patients. Furthermore, the expression of AIP1 was negatively correlated with HOMA-IR and WHR. The proteomic analyses showed that in the OAT of obese patients with T2D, inflammatory response was increased. Furthermore, we found that AIP1 deficiency in adipocytes significantly augmented the secretion of TNF-α, which was positively correlated with the T2D index HOMA-IR, *via* JNK and p38 MAPK/ERK signaling (**Figure 7**).

T2D is highly prevalent worldwide. Among the multiple causes of deaths, cardiovascular disease is the primary cause of mortality in individuals with T2D, and it accounts for 69.5% of deaths of individuals with diabetes (15, 24). Furthermore, multifactorial interventions that target cardiovascular risk factors can improve the management of diabetes (25). Previous studies have shown that AIP1 is involved in several cardiovascular diseases, including transplant graft arteriosclerosis (12), inflammatory angiogenesis (15), and atherosclerosis (16). In addition, a recent study reported that high glucose or high insulin levels would increase AIP1 expression in human endothelial cells (26). However, whether AIP1 is associated with T2D remains unclear. To the best of our knowledge, our study is the first to show that AIP1 was decreased in the OAT of patients with T2D and that AIP1 expression negatively correlated with HOMA-IR and WHR, indicating that AIP1 is a vital predictor of T2D.

There is an overwhelming amount of evidence revealing that inflammation has a causative role in T2D. The inflammatory genes and macrophage-specific genes are upregulated in white adipose tissue of patients with obesity, followed by an increase in circulating insulin levels, and they have been proposed to be linked to insulin resistance (10). Our study also revealed that the OAT from patients with T2D was in a state of inflammation. Studies have reported that TNF-α is elevated in the adipose tissue of patients with diabetes, and blocking TNF-α can alleviate insulin resistance *in vivo (* 27 *)*. We further confirm the increased expression of TNF-α in the OAT of patients with T2D, along with decreased AIP1 expression. Indeed, the underlying regulatory mechanism of TNF-α production remains unclear. Interestingly, we found that AIP1 deficiency in adipocytes increased the protein level of TNF-α, together with higher sugar level in the supernatant, indicating that AIP1 deficiency partially increases insulin resistance *via* TNF-α. However, further research is required to determine how AIP1 regulates TNF-α signaling pathways and its association with insulin resistance.

AIP1 is an ASK1-interacting protein that was recently identified to mediate TNF-α-induced activation of ASK1 by inducing the separation of ASK1 from its inhibitor 14-3-3 and then activating JNK and p38 MAPK signaling, which are downstream of ASK1 (11, 28). Knockout of ASK1 in Akita mice delays the development of diabetes. Furthermore, *in vivo* models of mouse obesity have demonstrated that the absence of JNK1 would improve insulin sensitivity (29). In addition, studies on Kyoto Encyclopedia of Genes and Genomes (KEGG) pathway analysis of T2D-associated genes have revealed that MAPK signaling pathways are associated with T2D (30, 31). An *in vivo* study using a p38 MAPK inhibitor to treat db/db mice showed that inhibition of the p38 MAPK pathway could reduce blood glucose level and improve the function of β-cells, thus ameliorating the pathogenesis of T2D (32). In line with the findings of these studies, our results showed that AIP1 deficiency in OAT would promote the TNF-α-induced activation of JNK and MAPK (p38 and ERK) signaling, and inhibition of these signaling using specific inhibitors would reverse the changes caused by AIP1 deficiency in adipocytes. This indicates that AIP1 plays a protective role in the formation of T2D partially *via* inhibition of JNK and MAPK (p38 and ERK) signaling.

Nonetheless, further studies are required to uncover the direct function of AIP1 in T2D using animal models. Besides, how AIP1 mediates JNK, p38 MAPK, and ERK signaling as well as insulin secretion, deserves further investigation.

## Conclusion

In conclusion, AIP1, a novel member of the Ras-GAP family, was decreased in the OAT of patients with T2D. Furthermore, AIP1 expression negatively correlated with HOMA-IR and WHR, the major features of T2D. Mechanistically, AIP1 deficiency in the OAT elevated the expression of TNF-α *via* JNK and p38 MAPK/ERK signaling.

## Data Availability Statement

The original contributions presented in the study are included in the article/supplementary files. Further inquiries can be directed to the corresponding authors.

## Ethics Statement 

The studies involving human participants were reviewed and approved by Jinshazhou Hospital of Guangzhou University of Traditional Chinese Medicine Ethics Committee. The patients/participants provided their written informed consent to participate in this study.

## Author Contributions

LH designed the project. ZS and CC performed the experiments together. LH, ZS, and CC wrote the manuscript. BL, JH, and WJ revised the manuscript. LW and WJ contributed to the reagents and provided financial support. All authors contributed to the article and approved the submitted version.

## Funding

This research was supported by the National Natural Science Foundation of P. R. China (NSFC, 81900412, 91939109, 81970420), Project funded by China Postdoctoral Science Foundation (2019M653194), Science and Technology Planning Project of Guangdong Province, China (2019B030316024, 2018A0303130338), and Guangzhou Science and Technology Planning Project (202002020069, 20190410081).

## Conflict of Interest

The authors declare that the research was conducted in the absence of any commercial or financial relationships that could be construed as a potential conflict of interest.

## Publisher’s Note

All claims expressed in this article are solely those of the authors and do not necessarily represent those of their affiliated organizations, or those of the publisher, the editors and the reviewers. Any product that may be evaluated in this article, or claim that may be made by its manufacturer, is not guaranteed or endorsed by the publisher.
