# Corrigendum: ASK1-interacting protein 1 acts as a novel predictor of type 2 diabetes

**DOI:** 10.3389/fendo.2022.981231

**Published:** 2022-08-16

**Authors:** Zhigao Song, Cong Chen, Jipei He, Bixia Liu, Weidong Ji, Liangping Wu, Li He

**Affiliations:** ^1^ Center for Translational Medicine, The First Affiliated Hospital, Sun Yat-sen University, Guangzhou, China; ^2^ Department of Cardiovascular Surgery, Zhujiang Hospital of Southern Medical University, Guangzhou, China; ^3^ Department of Metabolic Surgery, Your Doctor Medical Group, Guangzhou, China; ^4^ Department of Metabolic Surgery, Jinshazhou Hospital of Guangzhou University of Traditional Chinese Medicine, Guangzhou, China; ^5^ Medical Research Center, Sun Yat-sen Memorial Hospital, Sun Yat-sen University, Guangzhou, China

**Keywords:** AIP1, visceral adipose, adipose inflammation, insulin resistance, type 2 diabetes

## Error in author list

In the published article, there was an error in the author list, and author Weidong Ji ^1^ was erroneously cited. The corrected author list appears below.

Zhigao Song ^1,2†^, Cong Chen ^1†^, Jipei He ^3^, Bixia Liu ^1^, Weidong Ji ^1*^, Liangping Wu ^3,4*^ and Li He ^1,5*^

The authors apologize for this error and state that this does not change the scientific conclusiosns of the article in any way. The original article has been updated.

## Publisher’s note

All claims expressed in this article are solely those of the authors and do not necessarily represent those of their affiliated organizations, or those of the publisher, the editors and the reviewers. Any product that may be evaluated in this article, or claim that may be made by its manufacturer, is not guaranteed or endorsed by the publisher.

